# Exploring the Impact of Early Decisions in Variable Ordering for Constraint Satisfaction Problems

**DOI:** 10.1155/2018/6103726

**Published:** 2018-02-22

**Authors:** José Carlos Ortiz-Bayliss, Ivan Amaya, Santiago Enrique Conant-Pablos, Hugo Terashima-Marín

**Affiliations:** Tecnológico de Monterrey, Escuela de Ingeniería y Ciencias, Ave. Eugenio Garza Sada 2501 Sur, Col. Tecnológico, 64849 Monterrey, NL, Mexico

## Abstract

When solving constraint satisfaction problems (CSPs), it is a common practice to rely on heuristics to decide which variable should be instantiated at each stage of the search. But, this ordering influences the search cost. Even so, and to the best of our knowledge, no earlier work has dealt with how first variable orderings affect the overall cost. In this paper, we explore the cost of finding high-quality orderings of variables within constraint satisfaction problems. We also study differences among the orderings produced by some commonly used heuristics and the way bad first decisions affect the search cost. One of the most important findings of this work confirms the paramount importance of first decisions. Another one is the evidence that many of the existing variable ordering heuristics fail to appropriately select the first variable to instantiate. Another one is the evidence that many of the existing variable ordering heuristics fail to appropriately select the first variable to instantiate. We propose a simple method to improve early decisions of heuristics. By using it, performance of heuristics increases.

## 1. Introduction

Constraint satisfaction problems (CSPs) are combinatorial problems classified inside the NP-Complete class [1]. Their importance has boomed throughout the years, since there is a wide range of practical applications in artificial intelligence and operational research that can be represented as CSPs [2–5].

In a general sense, a CSP [6] can be defined by a sequence of variables, *V*. Each variable in this set, *v* ∈ *V*, can be assigned a finite set of available values *D*_*v*_ (its domain). Moreover, a set of constraints *C* restricts the feasible combinations of values that can simultaneously occur. Solving a CSP requires assigning a feasible value to every variable in such a way that all constraints are satisfied [7]. Each assignment is commonly referred to as an “instantiation,” and both terms are used indistinctly throughout this manuscript. A commonly used solution approach for CSPs relies on a search tree representation which is explored in a depth-first manner. In this search tree, every node represents the assignment of one specific variable. However, afterwards (i.e., after arriving at a node) constraints must be checked to verify that the solution is still feasible. Thus, the total number of verifications, known as consistency checks, can be used as a performance metric since a lower number represents a better assigning procedure. Should an assignment break one or more constraints, a different value must be considered for that variable. Moreover, if a variable runs out of values to be assigned, a previous assignment must be changed. This process is known as backtracking [8] and represents the basis for any search-tree-based method for solving CSPs. In practice, assigning variables in different orders changes the cost of the search since the search space is explored through a different path. For this reason, it is paramount to incorporate techniques for ordering variables appropriately.

A CSP instance with *n* variables has *N*_*o*_ = *n*! different ways in which variables can be ordered. The number of solutions depends on the size of the domain of each variable, |*D*_*v*_|. Among all existing orderings, only a few will optimize the search and minimize its cost. Unfortunately, due to the huge search space, it is not feasible to explore the whole space of orderings to find the best ones. For example, for large values of *n*, the number of solutions, given by *N*_*s*_ = ∏_*v*∈*V*_|*D*_*v*_|, is surpassed by the number of orderings; that is, *N*_*o*_ ≫ *N*_*s*_. From now on, we will use the terms* ordering* and* permutation* of variables indistinctly. In this work, we use these terms to refer to an ordered sequence of variables, where no repetitions are allowed. When a CSP instance is to be solved, the first variable in the permutation is instantiated first. Conversely, the last variable in the permutation is the last to be assigned a value. Different permutations of variables represent different costs of the search.

Authors have shown time and again that a single solver cannot tackle every CSP instance in the best possible way [9, 10]. Thus, research efforts have migrated from improving from specific techniques to finding ways in which they can be best combined. A classical example of the eventual benefit of doing so is the use of algorithm portfolios such as SATzilla [11], for SAT, and CP-Hydra [12] for CSPs. Algorithm portfolios analyze each problem and decide which among the available solvers should handle the task. Another solution is Instance-Specific Algorithm Configuration (ISAC) [13]. Though similar in nature to algorithm portfolios, ISAC differs since it changes the configuration of a given solver. Another related concept is autonomous search [14, 15], which dynamically adapts the variable/value ordering during the search, according to search performance indicators. Hyperheuristics are another example of methods that adapt to the current state of the search in order to work with the best available solving strategies. Hyperheuristics do not operate on the space of solutions (thus dealing with the problem directly). Instead, they operate on the space of solvers by identifying when it is recommended to change to a different solver (even if a solution process is currently underway). There are, of course, many and varied ways in which a hyperheuristic can be constructed. Some of the most recent and direct examples take into account filtering of heuristics that will be considered during the process (thus discarding redundant ones) [16]. Another example transforms the features that identify a given state of the problem to improve the quality of the feature space [17].

The task of solving a CSP is not to find a “good” ordering of variables, but to find a solution to the instance. Moreover, this solution also includes concluding that the instance is unsatisfiable. Besides, performance of a solver is linked to the cost of the search (usually measured in time, expanded nodes, or consistency checks). Thus, in this paper we analyze the order of variable selections as a mean to minimizing the cost of the search. We use this approach to find a path that leads to a solution within an acceptable consumption of resources. Therefore, we analyze how changes in the orderings of variables (particularly on the first variables in the permutations) affect the cost of the search. We obtain evidence showing that it may be enough to just analyze a few of the first variables in a permutation. Based on the costs of other permutations for the same instance, we can estimate whether the complete ordering is likely to produce a solution or not. We also test a set of well-known variable ordering heuristics and show that they do not necessarily make the right decisions at early stages of the search. By using the information obtained from this investigation, we proposed a simple method to help heuristics decide on their early decisions, so they improve their performance. In summary, the main contributions of this investigation are as follows:

- A deep analysis of the orderings produced by seven commonly used variable ordering heuristics for CSPs and their impact on the cost of the search
- Experimental evidence supporting the empirical idea that early decisions made during the search are more important than latter ones with regard to the cost of the search
- Experimental evidence suggesting that the variable ordering heuristics analyzed in this work can be improved by focusing on their early decisions
- A first approach on a subproblem-sampling method that improves the way heuristics work by helping them choose a more suitable variable to start the search with.

This paper is organized as follows. A revision of related investigations and existing variable ordering heuristics is presented in Section 2.  Section 3 presents information regarding the sets of instances analyzed in this work, as well as an overview of the testing that was carried out.  Section 4 presents the experiments related to analyzing different random permutations of variables. Here, we focus on how small changes in such permutations impact the cost of the search, on both random and real-world instances. Data regarding experiments about early decisions of various well-known variable ordering heuristics are provided in Section 5. This section also presents the description of the subproblem-sampling method for improving early decisions of variable ordering heuristics. Finally, Section 6 presents the conclusion and future work derived from this investigation.

## 2. Background and Related Work

In a CSP, the way values are assigned to a variable affects the cost of the search. Therefore, improving variable ordering in CSPs may lead to reduced costs. This idea has motivated the generation and review of a large number of ordering strategies [18–21]. Specifically, various heuristics have been proposed to dynamically construct the ordering of variables as the search progresses, instantiating one variable at a time. The complexity of heuristics varies according to the features they analyze. For example, DOM [22] is a widely used heuristic that prioritizes the variable with the smallest domain. DEG [23] is also one commonly used heuristic that prefers the variable with the largest degree. Such degree is calculated as the number of edges incident to that variable. More complex heuristics combine the criteria of other simpler heuristics to improve their decisions. For example, DOM/DEG [24] prefers the variable that minimizes the quotient of the domain size over the degree of the variable. Other heuristics take advantage of a more detailed description of the problem state that is usually more expensive to estimate. For example, heuristics RHO and *K* [25] analyze the solution density and *κ* factor of the instances, respectively. These two features provide a more detailed description of the instances, but they usually require more time to be computed. One of the most recent developments on variable ordering heuristics applies the learning-from-failure approach which includes heuristics such as WDEG [26, 27] and DOM/WDEG [26]. These heuristics are referred to as conflict-driven heuristics because they store and exploit information about previous failures by weighting the constraints in the instances they solve.

### 2.1. Heuristics Considered in This Work

Seven commonly used variable ordering heuristics have been considered for this investigation. In the case of ties, the lexical ordering of the identifiers of the variables is used to select the next variable to instantiate. Although some of them were first described more than two decades ago, they remain useful and competitive nowadays for various benchmark instances. To sum up, the variable ordering heuristics used in this work are described as follows:

(i) * DOM*. This heuristic instantiates first the variable that is more likely to fail. DOM estimates how likely a variable is to fail by counting the number of values in its domain [22, 28, 29], and chooses the variable with the fewest available values.
(ii) * DEG*. It considers the degree of the variables to decide which one to instantiate before the others. Thus, DEG selects the variable with the largest degree [23, 28, 30].
(iii) * DOM/DEG*. It is the result of the combination of DOM and DEG into a single heuristic. DOM/DEG tries first the variable with the smallest quotient of the domain size over the degree of the variables [31].
(iv) * RHO*. This heuristic is based on the calculation of *ρ*, which represents the approximated solution density of the CSP instance [32]. RHO instantiates first the variable that maximizes the fraction of solution states of the resulting instance:
  (1) $$\begin{array}{c} \rho =\underset{c\in C}{\prod }\left(\;1-p_{c}\;\right), \end{array}$$
where *p*_*c*_ represents the tightness of constraint *c* (the proportion of forbidden pairs of values in the constraint).
(v) *K*. This heuristic selects first the variable that minimizes the *κ* value of the resulting instance [25]:
  (2) $$\begin{array}{c} \kappa =\frac{-\sum_{c\in C}^{}{log}_{2}\left(1-p_{c}\right)}{\sum_{v\in V}^{}{log}_{2}\left(\left|D_{v}\right|\right)}, \end{array}$$
where |*D*_*v*_| is the domain size of variable *v*.
(vi) * WDEG*. This heuristic attaches a weight to every constraint of the problem [26, 27]. The weights are initialized to one and increased by one whenever the respective constraint corresponding to the weight being affected fails during the search. Then, the weighted degree of a variable is calculated as the sum of the weights of the constraints in which the variable is currently involved. WDEG gives priority to the variable with the largest weighted degree.
(vii) * DOM/WDEG*. DOM/WDEG is a revision of DOM and WDEG. This heuristic selects first the variable that minimizes the quotient of the domain size over the weighted degree of the variable [26].

Similarly, several value ordering heuristics exist [33, 34]. Nonetheless, in this work values are always assigned following the MINC heuristic [35] because of its simplicity. MINC selects the values in descending ordering, based on the number of conflicts in which they participate.

Some of the heuristics considered in this work were initially proposed as static approaches (i.e., they order variables and values before entering the solving process). In spite of this, we implemented all of them as dynamic ordering heuristics. Thus, all heuristics order variables and values as the search takes place. Because of this, they can take into account changes made to the instance as the result of previously assigned variables.

## 3. Methodology

We have mentioned that the space of permutations of variables is huge. Among all the possible orderings, how do we define a good one? A good ordering is a permutation of the *n* variables such that when the variables are assigned in that order, the cost of the search is below some critical value we consider acceptable. Since any assignment of variables is acceptable as long as it is feasible (every variable is assigned a value and all the constraints are satisfied), the cost of the search refers to the number of consistency checks required to find the first solution and not to the quality of the solution itself. Every time a constraint is revised, given the current values assigned to the variables involved, a consistency check occurs. The larger the number of consistency checks during the search, the higher the cost of solving the instance. Because the ordering of the variables for assignment affects the cost of the search [36], we will also use the cost of the permutation to refer to the cost of the search when such permutation is used to instantiate the variables. Thus, we can define an optimal ordering as a permutation of variables such that when the variables are instantiated in that order, a solution is found or the instance is proven unsatisfiable by using the fewest consistency checks. Because it is possible that two or more permutations have the same cost, there are cases where more than one permutation is considered optimal for a given instance. As it is not feasible to exhaustively enumerate all the orderings of variables for an instance, the optimal ordering is defined only with respect to the other orderings evaluated. We cannot guarantee that the optimal ordering will still be the best one when compared against other permutations in the unexplored regions of the space. Thus, when we refer to optimal permutations in this investigation, we refer only to local optimal ones.

In this investigation, the solver used for all the experiments is fully implemented in Java and incorporates MAC (maintaining arc consistency) and backjumping to speed up the search. To avoid extremely long runs, we impose a time limit for the solver to work on the instances. In all cases, the search stops if the solver exceeds the time limit while working on an instance. In this work, the time limit is defined for each individual experiment. Although we are aware that restarting the search is a useful strategy for avoiding being stuck with bad initial decisions [37], the solver used in this investigation does not incorporate such a strategy, since we are interested in exploring the way in which good (and bad) first choices affect the overall cost of the solving process.

The following sections present the main information related to the tests carried out throughout this work. The first part presents everything related to the different sets of instances that were used. The second one presents an overview of the testing carried out.

### 3.1. Set of Instances Analyzed in This Work

Before moving forward into this investigation, we describe the instances studied. All the instances used in this investigation are binary, as they contain only constraints that involve two variables, and with constraints represented in extension. When needed, the instances in this investigation are characterized by two standard CSP features: the constraint density (*p*_1_) and the constraint tightness (*p*_2_). The constraint density indicates the ratio of constraints within the instance: 2 | *C*|/*n*(*n* − 1), where *n* and |*C*| stand for the number of variables and the number of constraints in the instance, respectively. The closer the value of *p*_1_ to 1, the larger the number of constraints within the instance. The constraint tightness, on the other hand, represents the average ratio of conflicts within the constraints: (1/|*C*|)∑_*c*∈*C*_*p*_*c*_, where *p*_*c*_ represents the proportion of forbidden pairs of values in constraint *c*. In the case of binary CSPs, a conflict is a pair of values 〈*x*, *y*〉 that is not allowed for two variables at the same time. The larger the number of conflicts, the more likely an instance is unsatisfiable.

This work considers both randomly generated instances and structured ones taken from public sources. The random instances used for the first experiment were generated by using random generation model B [38], while the rest of the randomly generated instances were produced by using model RB [39]. Model B produces a graph with exactly (*p*_1_*n*(*n* − 1))/2 constraints. Later, for each constraint, it creates exactly *p*_2_|*D*_*u*_||*D*_*v*_| pairs of conflicting values among variables *u* and *v*, where |*D*_*u*_| and |*D*_*v*_| represent the domain sizes of variables *u* and *v*, respectively. Model RB is a revision of model B that requires four parameters to describe the instances produced: *n*, to indicate the number of variables; *α*, to determine the domain size (given by *n*^*α*^); *r*, to estimate the number of constraints (given by *r*log⁡*n*); and *p*, to specify the tightness of each constraint. The idea behind using two generation models is that the generation parameters of model B are suitable for producing grids of instances with specific values of *p*_1_ and *p*_2_, which was something we required for our first experiment. Model RB, on the other hand, has been commonly used to produce hard satisfiable instances [40] like the ones we needed for the rest of the experiments with randomly generated instances.

There is a relation between the structure of CSPs and the difficulty of solving them with search algorithms [41]. Specifically, the median cost of many search algorithms for CSP exhibits an abrupt change in the probability that an instance has a solution around a specific value of a structural parameter [41]. The region where this phenomenon occurs is known as the phase transition. Close to the phase transition, the solvable problems have very few solutions and the algorithm must, on average, explore a larger region of the search space before finding a solution [42]. To measure the hardness of the instances (and to indirectly decide how far they are from the phase transition region), we used the *κ* value [25] (see (2)). This concept is suggested as a general measure of how restricted a combinatorial problem is. If *κ* is small, the problems usually have many solutions with respect to their size. Conversely, when *κ* is large, the problems often have few solutions or none at all. Difficult problems, the ones inside the phase transition region, occur when *κ* ≈ 1.

The instances considered for this investigation are organized in the following sets:

- *RAND-A*. The instances in this set were generated by using model B and are uniformly distributed on the space *p*_1_ × *p*_2_. The space of instances *p*_1_ × *p*_2_ was segmented by steps of 0.05 on each axis, resulting in a grid of 20 × 20 cells. Inside each cell, 10 random instances with 25 variables and 15 values in their domains were generated. The values of *p*_1_ and *p*_2_ were randomly chosen for each instance according to the boundaries of the respective cell. A total of 4000 random instances are included in this set.
- *RAND-B*. This set contains 200 satisfiable randomly generated instances through model RB and considered hard to solve according to the *κ* criterion [25]. To produce these instances, the values of *n*, *r*, and *p* were randomly selected in the ranges [15,30], [0, *n*(*n* − 1)/2log⁡(*n*)], and [0,1], respectively. To guarantee that only hard satisfiable instances were included in the set, two conditions were revised. First, we revised the value of *κ* (see (2)) of the resulting instance to guarantee that it was in the range [0.925,1.05] (the range was empirically defined based on previous experimentation). If the value was outside this range, the instance was discarded and a new one was generated and revised. The second verification concerns the instance being satisfiable. For this reason, we solved each instance with the seven heuristics described in Section 2. If at least one of the seven heuristics was able to solve the instance within a time limit of 30 seconds, the instance was included in the set. Otherwise, the instance was discarded and a new one was generated and revised. By following this methodology, we constructed set RAND-B which contains a mixture of hard satisfiable instances with different numbers of variables and domain sizes.
- *RAND-C*. This set contains 200 hard satisfiable instances produced with model RB. The methodology used to construct set RAND-B was also used to generate set RAND-C but, this time, only instances with 25 variables and 15 values in their domains were generated. As in set RAND-B, the values of *κ* for these instances lie in the range [0.925,1.05].
- *JOBSHOP*. These instances were taken from a public repository (http://www.cril.univ-artois.fr/~lecoutre/research/benchmarks/jobShop-e0ddr1.tgz, http://www.cril.univ-artois.fr/~lecoutre/research/benchmarks/jobShop-e0ddr2.tgz). The instances in set JOBSHOP contain 50 variables with nonuniform domains of at least 100 values. All the instances in this set are satisfiable.
- *DRIVER*. This set contains seven satisfiable real-world instances taken from a public repository (http://www.cril.univ-artois.fr/~lecoutre/research/benchmarks/driver.tgz). The number of variables varies among the instances, ranging from 71 to 605. The values in the domains of the variables are also different among the instances.
- *QCP-15*. This small set contains Quasi-group Completion Problem (QCP) instances, where some of them are unsatisfiable. In QCP, the task is to determine whether the remaining entries of the partial Latin square can be filled in such a way that we obtain a complete Latin square. This set contains instances with 225 variables and domains of different sizes. Set QCP-15 was taken from a public repository (http://www.cril.univ-artois.fr/~lecoutre/research/benchmarks/QCP-15.tgz).

### 3.2. Experiments Carried Out in This work

**Figure 1:**
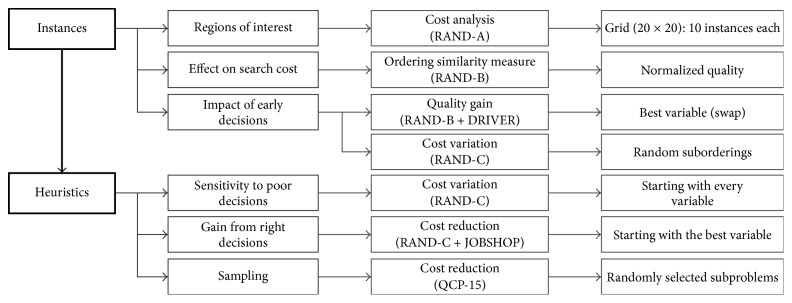
Two-part methodology followed throughout this work, with three stages each.

The focus of this work was twofold: analyzing the impact of early decisions of random orderings on different instances and analyzing how well standard heuristics deal with the problem of selecting the first variables to assign.  Figure 1 summarizes the way in which we split our experimental setting, which comprises six stages separated into two parts.

As an overview of the methodology, the setting is divided into two types of experiments. The first experiments are related to instances and how random and semirandom orderings of variables affect the cost of the search. In these experiments, we focus our attention on early decisions and how changes to the first variables of the permutations can improve the cost of the search. The second set of experiments analyzes standard heuristics and how sensitive they are to wrong initial decisions. We provide evidence that supports that these heuristics can significantly improve their performance if better decisions are made at early stages of the search. Finally, we propose a sampling strategy for helping heuristics to make better initial decisions.

The following sections describe the experiments conducted in this investigation as well as the main results obtained. Following the structure presented in Figure 1, the experiments are grouped into two sections.

## 4. Instance-Related Experiments

This section presents the results of the experiments related to random and semirandom permutations and how they affect the cost of solving different sets of instances.

### 4.1. Identification of Regions of Interest

As a first approach, we were interested in determining how the difficulty of a given problem varies as a function of its features. We can assume there exist regions where a large proportion of all possible orderings exhibit a low cost. For example, it is easy to see that for instances with many solutions almost any ordering will lead to a solution without much effort, thus making the order in which variables are instantiated virtually irrelevant. Similarly, for instances with few solutions it is likely that most orderings will find a solution only after a large number of consistency checks occur.

How difficult, then, is it to find a low-cost ordering of the variables to instantiate? To answer this question, we analyzed the effect of different orderings on the cost of solving the instances from set RAND-A (see Section 3.1 for details on this set). The instances in set RAND-A are distributed in a grid *p*_1_ × *p*_2_. Each cell in the grid contains 10 instances with 25 variables and 15 values in their domains. We measured the standard deviation of the costs of solving the 10 instances in each cell, resulting from using 30 different random permutations per instance. For this experiment, the maximum running time allowed for the solver to work on each instance was set to three seconds. The justification for this value is that we conducted some preliminary analysis on random instances similar to the ones used in this experiment and three seconds proved to be enough time for some permutations to allow the solver to complete such instances. Of course, not all the runs are expected to produce a solution, but in this work we are interested in showing that some initial decisions were more desirable than others. Thus, whether some orderings do not lead to a solution is also valuable information.

The idea behind this experiment was to estimate the likeliness of obtaining similar costs by using a random ordering of variables for a particular point (*p*_1_, *p*_2_). The smaller the standard deviation of the cell, the more likely it is to find permutations of variables with similar costs. In other words, by analyzing the standard deviation of the costs of different permutations, we can estimate how relevant the ordering of the variables is for certain regions of the space *p*_1_ × *p*_2_ and focus our efforts on those instances where the ordering influences the cost the most.

**Figure 2:**
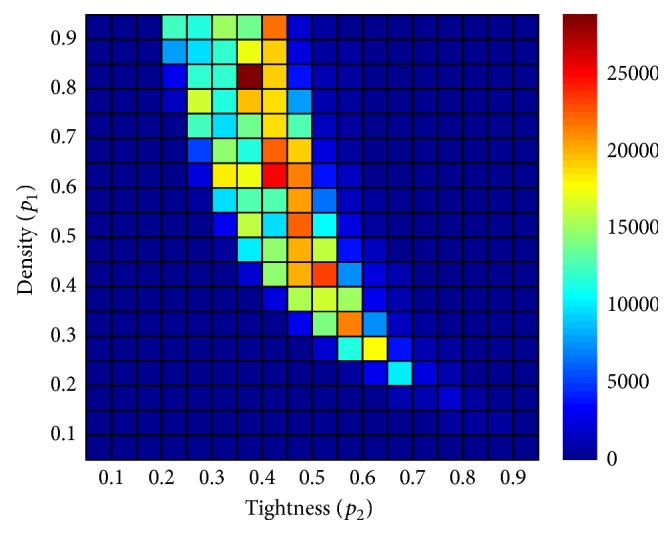
Standard deviation of the cost of different random orderings per cell in set RAND-A.

Figure 2 shows that the highest standard deviation of the costs generated by distinct random orderings of variables per cell in the grid of instances corresponds to the region dominated by difficult instances and usually referred to as the phase transition [41]. This is something reasonable because as we move away from this region, we find very easy satisfiable and unsatisfiable instances (ones are easy for having plenty of solutions while the others for having none), and any ordering in which the variables are instantiated seems to be equally competent. The further the instance is from the phase transition, the more likely it is that different orderings of variables produce similar costs. It is precisely in the region surrounding the phase transition where the highest deviations take place and where the order in which variables are instantiated becomes critical.

### 4.2. Effect of Decisions on the Search Cost

We have shown that the ordering in which the variables are assigned is more important for some instances than for others. The region in the instance space where the impact of the permutations of variables reaches its maximum is located, unsurprisingly, within the boundaries of the transition phase. In this experiment, we compared random permutations of variables and their costs by using the 200 hard satisfiable instances from set RAND-B (for details on this set, see Section 3.1). We deemed important to work only with satisfiable instances to guarantee that the *n* variables from each instance were included in every permutation. We solved each instance in the set with 30 different random permutations of variables and we saved the cost of the search by using each permutation. A total of 6000 permutations were tested as part of this experiment. For each instance, we calculated the normalized quality of each ordering by dividing the cost of using the best permutation for that instance over the cost of using each one of the remaining 29 permutations for the same instance. Then, the smaller the cost of the search with respect to the cost of using the best-known permutation, the higher the value of the normalized quality.

When we started working on this investigation, it seemed reasonable to think that low-cost permutations for a given instance would be similar to each other. Following this idea, we compared, for each instance, the best permutation against the remaining 29 for the same instance. The comparison of permutations *l*_1_ and *l*_2_ was done using a normalized version of Kendall's *τ* metric [43], calculated as shown in (3), where *P* is a pair of distinct elements in *l*_1_ and *l*_2_. ${\bar{K}}_{\left(i,j\right)}(l_{1},l_{2})=0$ if *i* and *j* are in the same order in *l*_1_ and *l*_2_ and it is equal to 1 if they are in the opposite order. Kendall's *τ* metric measures how different two permutations are, where 0 indicates that both permutations are exactly the same and 1 indicates that one is the inverse of the other.

(3) $$\begin{array}{c} \tau \left(\;l_{1},l_{2}\;\right)=\underset{\left(i,j\right)\in P}{\sum }{\bar{K}}_{\left(i,j\right)}\left(\;l_{1},l_{2}\;\right). \end{array}$$

**Figure 3:**
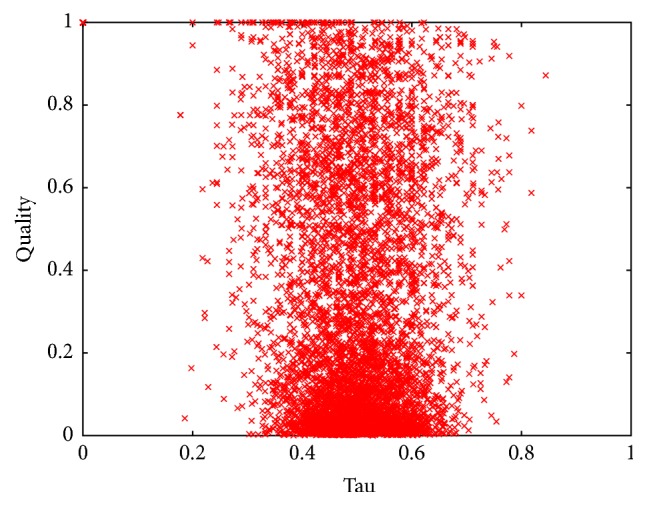
Normalized quality of the permutations used to solve the instances in set RAND-B against *τ* value (with respect to the best-known permutation of variables per instance).

Figure 3 depicts the normalized quality of each of the 6000 permutations analyzed in this experiment against the *τ* value obtained from the comparison between each permutation per instance and the best-known permutation for such instance.

According to our first ideas, we expected similar orderings of variables (characterized by small values of *τ*) to show similar costs for a particular instance. In other words, we expected that all the permutations with *τ* ≈ 0 for a particular instance would have a quality close to 1 on the same instance (as they would be similar to the best-known permutation for that instance). As we can observe from Figure 3, this is not the case. We detected a set of isolated points in (0,1) that correspond to the best permutations for each instance. Also, none of the permutations, in any case, was completely different from the best permutation per instance. This explains the lack of points with values of *τ* close to 1. We also observed some permutations with low values of *τ* but poor quality. The latter means that some permutations that are very similar to the best ordering for a given instance performed poorly when used on the same instance. This result was surprising, as we expected similar permutations to show similar costs. On the other hand, we detected permutations with large values of *τ* and high quality that correspond to the opposite case where very different permutations with respect to the best one for a given instance produced outstanding results. This result can be easily explained as there may be more than one ordering that minimizes the cost of the search for a particular instance. Thus, two different orderings may be good performers for a specific instance and present a high normalized quality (regardless of *τ* being close to 1). Conversely, similar permutations (the ones with very low values of *τ*) may show very distinct qualities because the position of a few variables in the permutation can impact the cost of the whole permutation but represent only small changes in the resulting value of *τ*.

### 4.3. Impact of Early Decisions

From the previous section, we learned that, according to *τ*, similar permutations of variables may exhibit different costs, even for the same instance. We also learned that different permutations can actually be good performers for the same instance. Then, what is the proper way to compare two permutations of variables and estimate how well they will perform on a particular instance? To answer this question, we observed the permutations obtained from the previous experiment and focused on the first variable in the best permutation per instance. We found that, for a given instance, the first variable in the best ordering was also located at the first positions of other good performing permutations (first to third positions of the permutations). This finding gave us the idea that it might not be necessary to compare complete permutations but a small subset of the first variables in the orderings to estimate how similar their costs are. This finding supports the empirical idea that the first variables in the ordering are critical to determine the cost of the search [44].

For this experiment, we focused on determining whether it is possible to compare orderings and estimate their costs using only the first variable in the permutations and on whether finding one suitable variable to start the sequence is enough to solve the CSP instance with a low cost. Aiming at answering these questions, we used the permutations and instances from the previous experiment (plus the instances from the set DRIVER) and defined a swap operator that exchanges one variable *x* with that on the first position of the permutation. For each instance, we identified the first variable, *x*_0_, of the best permutation and, for each of the 29 remaining permutations, we moved *x*_0_ to the beginning of the permutation via the previously defined swap operator. By doing so, we forced all 30 permutations of each instance to start with the first variable of the best permutation for that instance.

**Figure 4:**
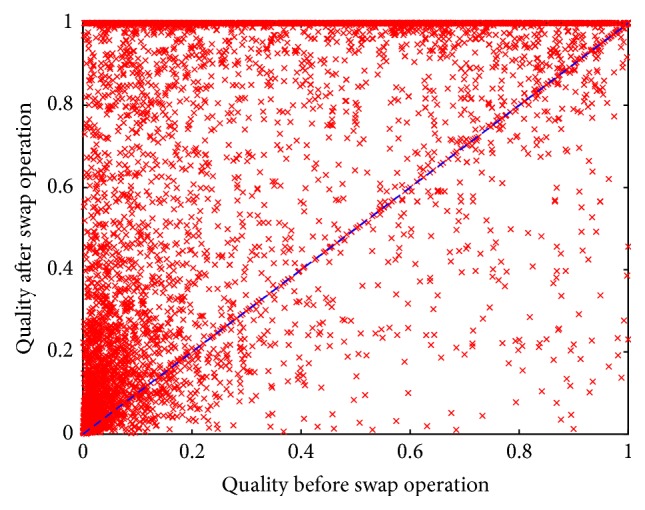
Normalized quality of the permutations used to solve the instances in set RAND-B (before and after the swap operation).

What we found from this simple swap operation (changing only one variable in each permutation) is that, in 79.78% of the instances, the quality of the permutations increased. In 7.98% of the orderings, the change resulted in normalized qualities above 1.0. This means that the improved orderings outperformed the best-known permutation for a particular instance (the one used to decide which variable to swap).  Figure 4 presents the change in the normalized quality of the permutations on the 200 hard instances from set RAND-B. The *x*-axis represents the quality before the swap operation, and the *y*-axis, the quality after the swap operation. The diagonal line represents the equation *x* = *y*, which serves as a reference: all the points above this line represent permutations whose quality was increased due to the swap operation, while points below the line represent permutations that reduced their quality as a result of the swap operation. For clarity, all the points with a quality larger than 1.0 after the swap operation have been plotted with a value of 1.0 in the *y*-axis.

We repeated the previous analysis on the instances from set DRIVER (see Section 3.1 for details on this set). As in the previous experiment, we analyzed the costs of the 30 random permutations per instance and identified the best one. Then, we compared the 29 remaining permutations per instance against the best ordering for that specific instance by using *τ* (see (3)). In the case of real-world instances, the time limit was increased to 30 seconds, as three seconds seemed to be insufficient for the solver to finish the search by using most of the permutations.

**Figure 5:**
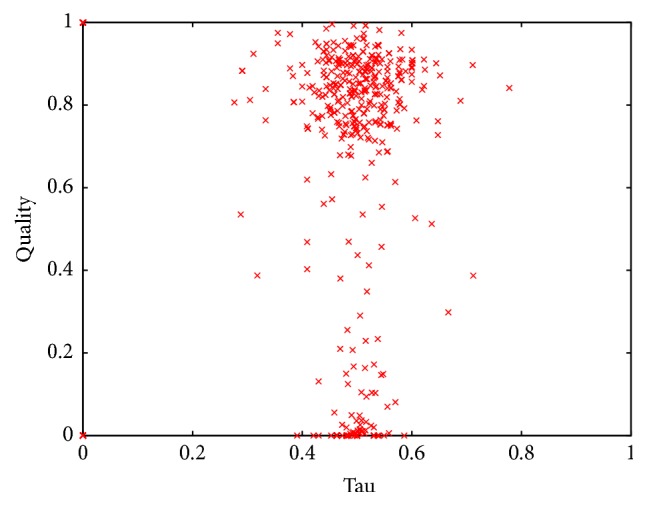
Normalized quality of the permutations used to solve the instances in set DRIVER against *τ* value (with respect to the best-known permutation of variables per instance).

Consistently with the results from the previous experiment, most of the permutations of variables used on set DRIVER present *τ* ≈ 0.5 (Figure 5) when compared against the best permutation per instance. We cannot observe a clear relation between the value of *τ* of two permutations and their quality. Then, as in the previous case, comparing all of the variables in the permutations seems to be an inefficient way to compare the qualities of two permutations of variables.

**Figure 6:**
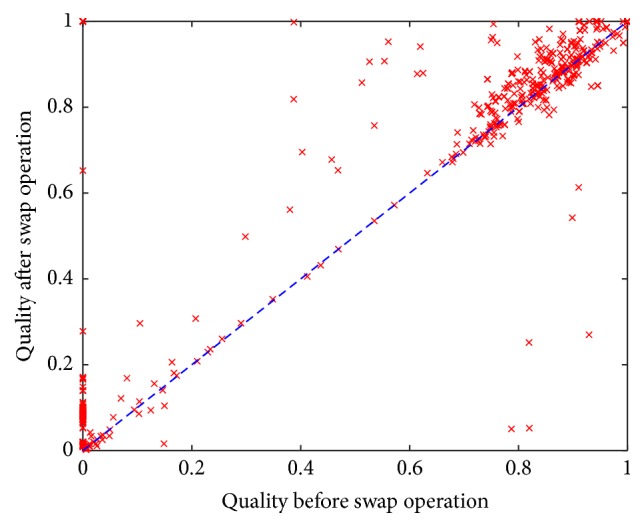
Normalized quality of the permutations used to solve the instances in set DRIVER (before and after the swap operation).

In the case of the instances contained in set DRIVER (Figure 6), the swap operation improved the quality in 67.24% of the permutations. In 6.18% of the cases, the quality improved above 1.0 after the swap operation. This means that, as in the instances from set RAND-B, for set DRIVER some modified permutations were even better than the best-known permutation for a particular instance before the swap operation. Also remarkable is the fact that, before the swap operation, the solver was unable to solve 19.9% of the instances in set DRIVER within the time limit. After the swap, all the runs solved their respective instance. These results provide experimental evidence that supports that the selection of the first variable to instantiate in a CSP is critical, even on larger real-world instances like the ones used in this experiment.

In an additional experiment, we analyzed how the first variable in the permutations affects the cost of the search. The idea here is to confirm if focusing our efforts on correctly selecting the first variable may be sufficient to increase the probabilities of minimizing the cost of the search. For these experiments, we used set RAND-C (see Section 3.1 for more details on this set). All the instances in this set were solved by using a semirandom heuristic (SRAND) that works as follows: SRAND is provided with a variable and a CSP instance to solve. Then, SRAND starts the search by instantiating the provided variable, while the remaining ones are selected at random. Thus, by using SRAND we can produce semirandom orderings of variables: permutations that start with one specific variable but where the remaining *n* − 1 variables are randomly ordered. For each of the 25 variables in every instance, 20 semirandom orderings were produced, totalling 500 runs per instance. Moreover, for each run we recorded two metrics: the number of consistency checks required by the search and the first variable in the permutation.

**Figure 7:**
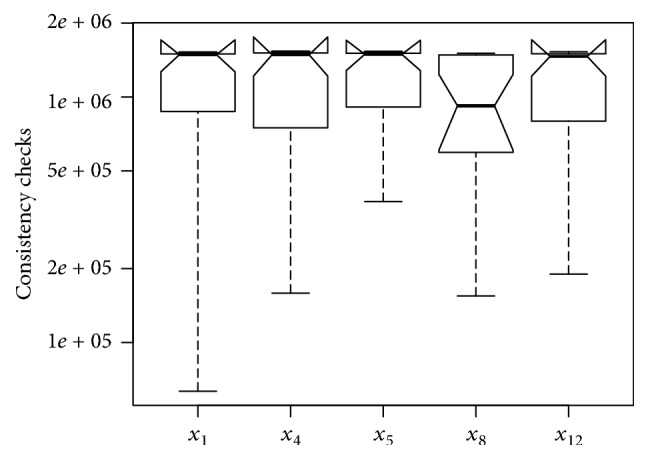
Costs of semirandom orderings per initial variable in the permutation for the first instance in set RAND-C.

Figure 7 depicts the boxplot with the cost of the 20 semirandom permutations on the first instance of set RAND-C (similar results are also observable on other instances in this set). The *x*-axis indicates the initial variable of the semirandom orderings and the *y*-axis indicates the cost of the search when a particular variable is used at the very first position of the permutation. For clarity, only permutations that finished the search in at least one of the 20 runs are shown in the figure. Based on the information depicted in Figure 7, we can conclude that, among the 25 possible initial variables in the orderings, assigning *x*_8_ first is the best option for this specific instance, as it obtains the lowest median cost in terms of consistency checks. Although the information suggests that five variables can be used as start point of the search (*x*_1_, *x*_4_, *x*_5_, *x*_8_, and *x*_12_), the statistical evidence suggests, with 95% of confidence, that *x*_8_ has a lower median cost than any of the other variables analyzed for this instance.

If the first variable in the ordering makes no difference, we would expect that almost all semirandom orderings produce similar costs, which is not the case for the first instance in set RAND-C. Although we have only shown the results for one instance in set RAND-C, this behaviour holds for the rest of the instances in the set. Only a few of the variables, when used at the beginning of the permutations, allow the solver to find a solution in the allocated time. Among the permutations that lead to a solution, only a few are statistically better than the others.

So far, we have only confirmed the empirical idea that not all the decisions are equally important regarding variable ordering. We have evidence that supports the idea that the first variable to instantiate is one of the most, if not the most, crucial decisions when solving a CSP by using a backtracking-based method. Our results suggest that the first variable in the permutation drastically increases or decreases the cost of the search. The next question to solve in this investigation is directly related to existing heuristics. We have pointed out the importance of the first variable, but are existing heuristics able to find a good starting point for the search? We will discuss this in the following experiments.

## 5. Heuristics-Related Experiments

This section continues the analysis of the impact of the first variable in the permutations but now focuses on some existing heuristics. We analyze the cost of the search derived from using the orderings obtained through different heuristics by studying the initial variables in such orderings. We analyze how decisions made at early stages of the search affect the performance of these heuristics in latter stages.

### 5.1. Sensitivity to Poor Decisions

Let us suppose that a given heuristic makes a bad decision at the very beginning of the search. A desirable characteristic of such heuristic would be that it could somehow use the upcoming choices of variables to compensate for the poor initial guess. If this were the case, no matter which variable was used at the beginning of the permutation, the search would require a similar cost. Unfortunately, the empirical evidence suggests that heuristics are vulnerable to a wrong initial choice of variable and that changing this specific variable has tremendous consequences in the cost of the search. Therefore, in this experiment we analyzed how the search guided by the heuristics described in Section 2 responds to changes on the starting variable (i.e., the one at the beginning of the permutation).

All the instances in set RAND-C were solved by using the seven heuristics described in Section 2 and the cost of the search was recorded for each heuristic on every instance in the set. Additionally, we propose modified versions of the heuristics described in Section 2. These modified versions of the heuristics are called DOM′, DEG′, DOM/DEG′, RHO′, K′, WDEG′, and DOM/WDEG′. All the modified versions of the heuristics work as follows: among all the variables in the instance, one is arbitrarily selected to start the search with. Once this variable has been instantiated, the rest of the ordering is defined by the criteria of each particular heuristic. For example, DOM′(*x*_0_) indicates that the first variable to assign is *x*_0_ (regardless of its domain size) but the remaining 24 variables will be instantiated according to DOM. Thus, we can force any of the traditional heuristics to start the search with one particular variable of our choice and then continue ordering variables in the standard fashion. Every instance in set RAND-C was solved 25 times with each modified heuristic. At each run, the initial variable was changed starting from *x*_0_ up to *x*_24_. The rest of the ordering was constructed by using the respective heuristic. For each run, we kept the initial variable in the permutation used for solving the instance and the cost obtained by using such permutation. These results are analyzed in the following experiments.

For each heuristic, we calculated the standard deviation of the cost of the search by using each of the 25 variables at the first position of the orderings. The higher the standard deviation per instance, the more impact the choice of the initial variable has on the cost of the search when using one specific heuristic. In other words, the standard deviation of the costs is used to estimate how sensitive heuristics are to the choice of the first variable to instantiate.

**Figure 8:**
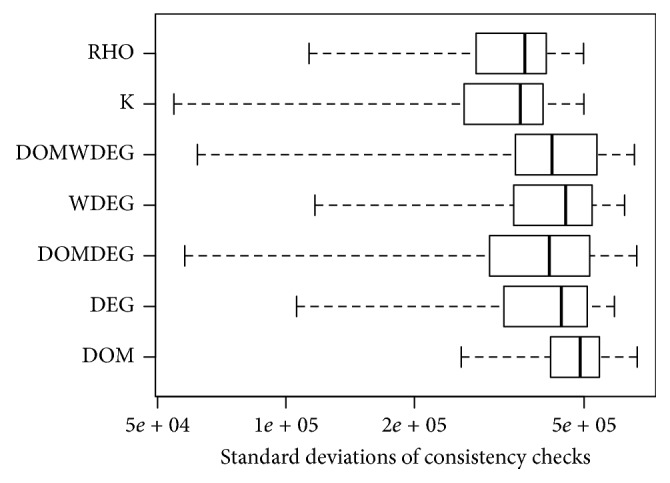
Sensitivity of the heuristics to the first decisions in set RAND-C.

Figure 8 shows the boxplot with the standard deviations of each heuristic in the 200 instances in set RAND-C. Among the seven heuristics, RHO and *K* are less sensitive to changes in the first variable (with 95% of confidence). On the contrary, changing the initial variable has a larger effect on DOM than in the rest of the heuristics.

### 5.2. Gain from Making the Right Decision

The experiments described in this section focus on the orderings obtained by commonly used heuristics and how they can be improved. The analysis is restricted to the first variables in the orderings, as we observed that they require most of our attention. Should we use the best variable to start the permutation (which is only known after we have tried all of them), we would obtain improved versions of the existing heuristics. For the purpose of this work, these new heuristics have been named DOM*∗*, DEG*∗*, DOMDEG*∗*, WDEG*∗*, DOMWDEG*∗*, K*∗*, and RHO*∗*. For example, DOM*∗* is equivalent to DOM (*x*), where *x* is the most suitable variable to start the search. This batch of tests considered two sets of instances: RAND-C and JOBSHOP.

**Figure 9:**
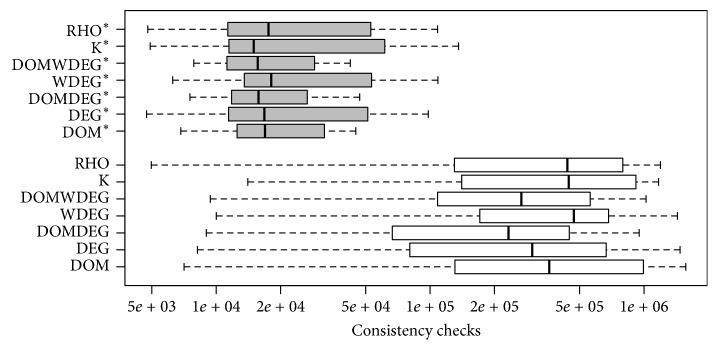
Performance of the heuristics in set RAND-C when forced to start the search with the first variable of the best-known permutation for each particular instance.

From the previous experiments, we were able to identify, for every heuristic, the best initial variable in the ordering for solving each instance in set RAND-C. As expected, this variable is not the same for all the instances.  Figure 9 shows the comparison of the performance of the existing heuristics and the performance of their improved versions in set RAND-C. However, it is important to remark that these findings represent a Utopian scenario, since a brute-force approach was used to test the effect of all variables so that the best one could be selected afterwards. Nonetheless, we consider this approach to be useful for showing the performance gap between the choice taken by the heuristic and the best possible choice. This paves the road for proposing a method that can identify a good enough variable in a cheaply enough fashion.

There is statistical evidence that supports that the modified versions of the heuristics are better than the standard ones. We ran a paired *t*-test for each pair of heuristics, where the alternative hypothesis was that the modified version of the heuristic had a lower mean than its standard version. In all the tests, the resulting *p* value was below 0.01. This provides strong evidence that the improved versions of the heuristics, the ones that make the right choice of the variable at the beginning of the search, outperform the standard ones.

**Figure 10:**
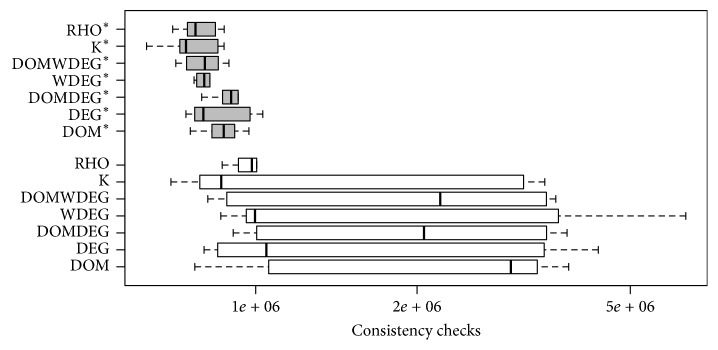
Performance of the heuristics in set JOBSHOP when forced to start the search with the first variable of the best-known permutation for each particular instance.

In order to demonstrate that this improvement is also present in non-randomly generated instances, we repeated the previous analysis on set JOBSHOP. The results shown in Figure 10 are similar to those observed on randomly generated instances but, this time, since the number of instances is small, the statistical evidence is not sufficient to state that all the improved versions of the heuristics outperform the standard ones. For this set of instances, DOM*∗*, DOMDEG*∗*, WDEG*∗*, DOMWDEG*∗*, and RHO*∗* are statistically better than their respective standard versions with a significance level of 5%.

Although the results presented in Figures 9 and 10 suggest that the seven heuristics analyzed can be improved, the information gathered as part of this experiment is only valid for the instances used to obtain such information. For this reason, we are aware of the need of a method capable of generalizing such information to be able to solve a wider set of instances.

### 5.3. Is It Possible to Improve How Heuristics Work?

So far, we have observed that it is not easy for standard heuristics to find the right variable to start the search with. For this reason, we propose a simple method that helps heuristics to make better decisions at the very first stages of the search. Our method differs from the well-known LDS [45–47] in the fact that we do not refute the decisions of the heuristics, but we use such heuristics on reduced instances in order to estimate which variable, once instantiated, is likely to reduce the cost of the search.

The method is based on an iterative sampling procedure that works as follows. For each iteration of the sampling process, a random subset *V*′ containing *n*′ variables is selected from the original set *V* in the instance. To select the random subset of variables, all the variables in the original instance are shuffled and then, the first *n*′ variables are selected. We then separate those variables from the original instance, creating a small subproblem that contains only *v* ∈ *V*′ variables (with their respective domains). The constraints that remain in the subproblem are those that only involve variables contained in *V*′ (any constraint that involves variables not contained in *V*′ is ignored). In this way, and because *n*′ ≪ *n*, the subproblems tend to be easier to solve than the original instance.

By using one particular heuristic (the one of interest at the moment), we solve each of the subproblems while recording the first variable chosen and the cost of the search, in this case the number of consistency checks. Each iteration produces and solves a different subproblem of *n*′ variables. When all the subproblems have been generated and solved, the method selects the variable that was used as starting point for solving the subproblem that required largest number of consistency checks. We then solve the original instance by using the respective modified heuristic (as depicted in Section 5.1). Basically, we solve the instance by instantiating the variable chosen by running a “quick test” on some random subproblems and then, we let one specific heuristic solve the rest of the instance.

We think that, by selecting the first variable instantiated when solving the subproblem that requires the most consistency checks, we are likely to find some difficulty in assigning variables (somehow related to the concept of* backdoors* [48]). In the original instance, solving first one of these variables is likely to represent a reduction in the cost of the search.

For this experiment, we solved set QCP-15, which proved to be particularly difficult for almost all the heuristics in this investigation. The time limit was again set to 30 seconds, as we did for harder sets of instances. For each one of the seven heuristics, we solved the whole set with that heuristic and run the sampling method 10 times. For each run, the method uses 1000 iterations and in each iteration a subproblem containing only five random variables is created and solved. All the values for the running parameters of the method were empirically defined.

**Figure 11:**
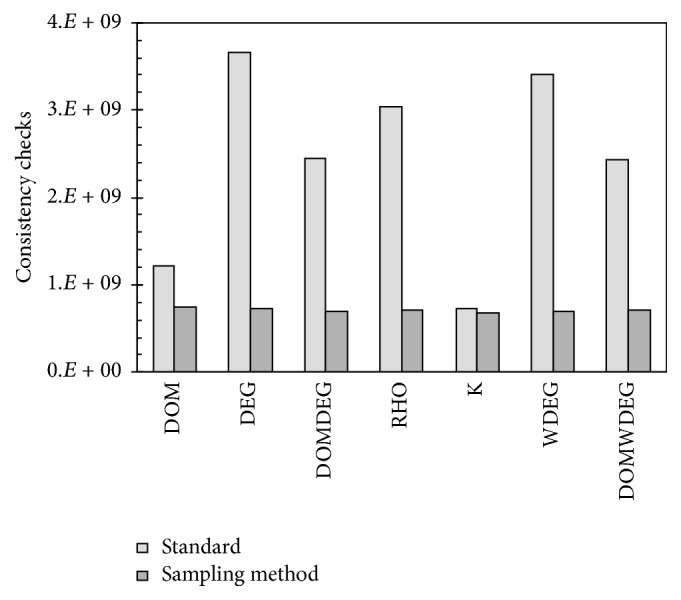
Cost of solving set QCP-15 when using each of the seven standard heuristics and the sampling strategy proposed.

Figure 11 shows the results of each heuristic on set QCP-15 and the average consistency checks of the 10 runs of the sampling method for each respective heuristic. The results of the sampling method include both the consistency checks consumed during the sampling process and the actual ones used when solving the original instances. The results show that the sampling method is, on average, capable of finding one suitable variable that helps the heuristics to improve their search. We can observe that, regardless of the heuristic used and its performance before using the proposed method, the sampling strategy tends to produce similar results, as good initial variables are selected for each heuristic. These good initial decisions, as shown in Section 5.2, dramatically improve the performance of existing heuristics.

It is important to stress the scope of applicability of our proposed approach. The sampling method has improved the quality of the variable ordering heuristics considered for this work. In fact, our evidence suggests that the sampling strategy tends to find “good” variables to start the search with. Nonetheless, we cannot guarantee that this behaviour propagates to all other heuristics. Thus, they may not be improved by using the same approach. The evidence we have gathered so far can only go as far as confirming the idea that some heuristics fail at selecting an adequate variable to start the search with. Therefore, by improving their early decisions, for example, by using the proposed sampling strategy, we can also improve their overall performance.

## 6. Conclusion and Future Work

An indirect way to reduce the cost of the search in CSPs is to find more reliable strategies to order the variables and their values as the search progresses. Studies about variable ordering found in the literature usually put the same effort for these decisions at all nodes of the search. However, we have found evidence suggesting that it would be better to focus on the first decisions. In most of the cases, a good decision at this point can significantly reduce the cost of the overall search.

By experimentally testing existing heuristics on different CSP instances, we found that they have plenty of room for improvement, especially if we focus on the first variable to instantiate. Our results apply to both random and real-world instances. It seems reasonable to think that the effect of first decisions lessens as the problem grows since there are more decisions to make. Although this makes sense, we were unable to confirm it. But, we found that first decisions are still critical for instances with more than 600 variables. Thus, our results seem to be unaffected by the growth of the instance size given by the increment in the number of variables.

In this work, we proposed a method for sampling the performance of heuristics on small random subsets of variables. This was done as an attempt to identify critical variables that should be instantiated before the others. We found evidence suggesting that this sampling approach is competitive and capable of improving the performance of the standard heuristics. This paper presented a preliminary version of our approach. A more refined version will be explored in future work.

An important consideration is that we have not tested the effect of changing the order in which the values for each variable are taken. We are aware that this may affect the cost of the search. But, we wanted to analyze the effect of variable ordering by itself, prior to including any other aspect to the analysis. We expect that including value ordering to the process will produce similar results to the ones obtained for variable ordering. But, this analysis is left for a future phase of this investigation.

One potential limitation of our approach might emerge when working with other types of constraints (e.g., *n*-ary constraints for large values of *n*). Although the solution model described in this work proved to be useful for CSPs containing binary constraints defined in extension, it may show a different performance if other types of constraints are considered. For example, if the constraints weakly propagate, then large domains of some variables will almost remain unaltered and the variable ordering strategies described in this work might not help. We consider this an important topic to be addressed as part of the future work.

Another important aspect that may be useful to explore in the future is how the branching scheme might alter our results. The branching scheme used in this investigation is usually referred to as *k*-way branching. Another classical scheme is 2-way branching. Through it, the search first assigns a variable with a value (left branch) before refuting the value from the domain of the variable (right branch).

Also, we have tested our ideas on satisfiable instances only to compare permutations that contain the same elements. We plan to extend our results to unsatisfiable instances. We will pursue a similar approach but using a modified Kendall's metric for unbalanced permutations. This unbalance may represent permutations with different number of elements or ones where some elements only appear in a couple of permutations.

We expect that the results obtained from this investigation lead us to more robust methods for variable ordering. Specifically, we want to simplify variable ordering for CSPs by generating ordering methods that focus on early decisions. This way, faster and cheaper ordering strategies can operate on the remaining variables. We want to use the information gathered from distinct permutations of variables to produce new ordering heuristics that exploit the information obtained so far. Unfortunately, at this point we are unsure of how to produce such heuristics. Thus, a further analysis of the instances and their first variables is required.

Finally, we are aware of the importance of hybrid approaches for solving hard CSPs. For this reason, we consider exploring the impact of the ideas described in this work on hybrid solvers such as algorithm portfolios and hyperheuristics. At the moment, we think that the strategies that are able to improve single variable ordering heuristics should also help improve more robust approaches, but we need to properly confirm this idea in future steps of this investigation.
